# A Case Report of Metastatic Retroperitoneal Angiosarcoma Mimicking Reactive Angioendotheliomatosis

**DOI:** 10.7759/cureus.80883

**Published:** 2025-03-20

**Authors:** Theodora Douvali, Georgios Sarris, Pelagia Pediaditi, Erasmia Adamou, Eleftheria Tampouratzi

**Affiliations:** 1 Department of Dermatology and Venereology, Andreas Syggros Hospital, National and Kapodistrian University of Athens, Athens, GRC; 2 Department of Dermatology and Venereology, Tzaneio General Hospital, Piraeus, GRC

**Keywords:** angiosarcoma, biological markers, histological mimicker, immunohistochemistry, reactive angioendotheliomatosis

## Abstract

Angiosarcoma is a malignant proliferation of vascular and lymphoendothelial origin. In terms of pathology outlines, this entity may resemble other growths derived from blood vessels. Reactive angioendotheliomatosis, an intravascular proliferation, is frequently hard to discern histologically from angiosarcoma. In this study, we outline a case initially presenting with features supporting reactive angioendotheliomatosis. Clinical progression and inconclusive histopathology raised suspicion, and further investigation was decided. Computed tomography imaging revealed a large retroperitoneal tumor, and subsequent surgery with additional histological examinations of the excised mass and cutaneous samples was performed, along with immunohistostaining. As a result, diagnosis and prognosis pointed to an entirely different direction.

## Introduction

Angiosarcoma is a rare malignant tumor of high aggressiveness that stems from lymphatic or vascular endothelial cells [1]. Due to the heterogeneity of angiosarcoma as a sarcoma subgroup, clinical occurrence comprises all anatomical areas, although less than 2% of soft tissue sarcomas are classified as angiosarcomas [1-3]. Most cases present with cutaneous involvement (around 60%), primarily on the head and neck, although visceral, osseous, retroperitoneal, and soft tissue may be affected [1,4].

Angiosarcoma has a predilection for adult and elderly populations, with a high frequency of local recurrence and metastatic disease [5]. Cases that present with clinical advancement or metastasis reach a percentage of 16%-44%, and overall survival ranges between six and 16 months after diagnosis [6]. The insight into angiosarcoma pathogenesis is not yet deepened, but risk factors such as chronic lymphedema, history of radiation, exposure to carcinogens (vinyl chloride, thorium dioxide, and arsenic), and certain genetic syndromes are well established [7]. While angiosarcoma evenly affects both sexes and may appear at any age, cutaneous angiosarcoma exhibits a clear preference for male patients from 60 to 71 years of median age [8].

Cutaneous manifestation of angiosarcoma may feature solitary or multiple bluish or red nodules with a tendency to ulceration and bleeding [7]. Lack of specificity of angiosarcoma symptoms hinders differentiation from other entities such as anaplastic melanoma and other epithelial carcinomas [1,9]. As a result, concerning angiosarcoma diagnosis, histological examination plays an important role, and immunohistochemistry is essential [5,10].

Given that prospective evidence is absent and angiosarcoma cases are scarce, optimization of treatment modalities is still a debatable subject. Currently, clinicians opt for surgery, radiotherapy, and chemotherapy with outcomes ranging widely, depending on tumor site, size, subtype, and resectability. Targeted therapy and immunotherapy have shown some promise in angiosarcoma treatment [11]. In this case report, we study an angiosarcoma case that was initially histopathologically diagnosed as a benign condition, reactive angioendotheliomatosis.

## Case presentation

**Figure 1:**
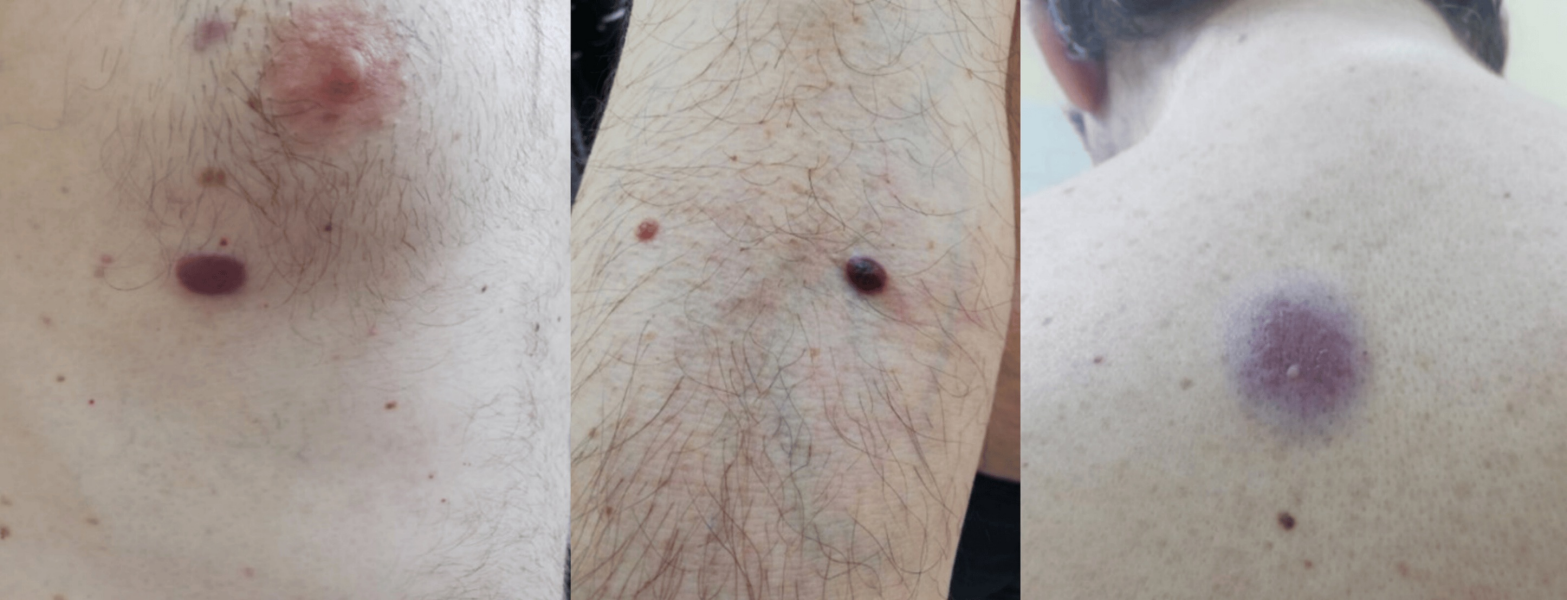
Diffuse red-purple plaques on the patient's trunk and arm

**Figure 2:**
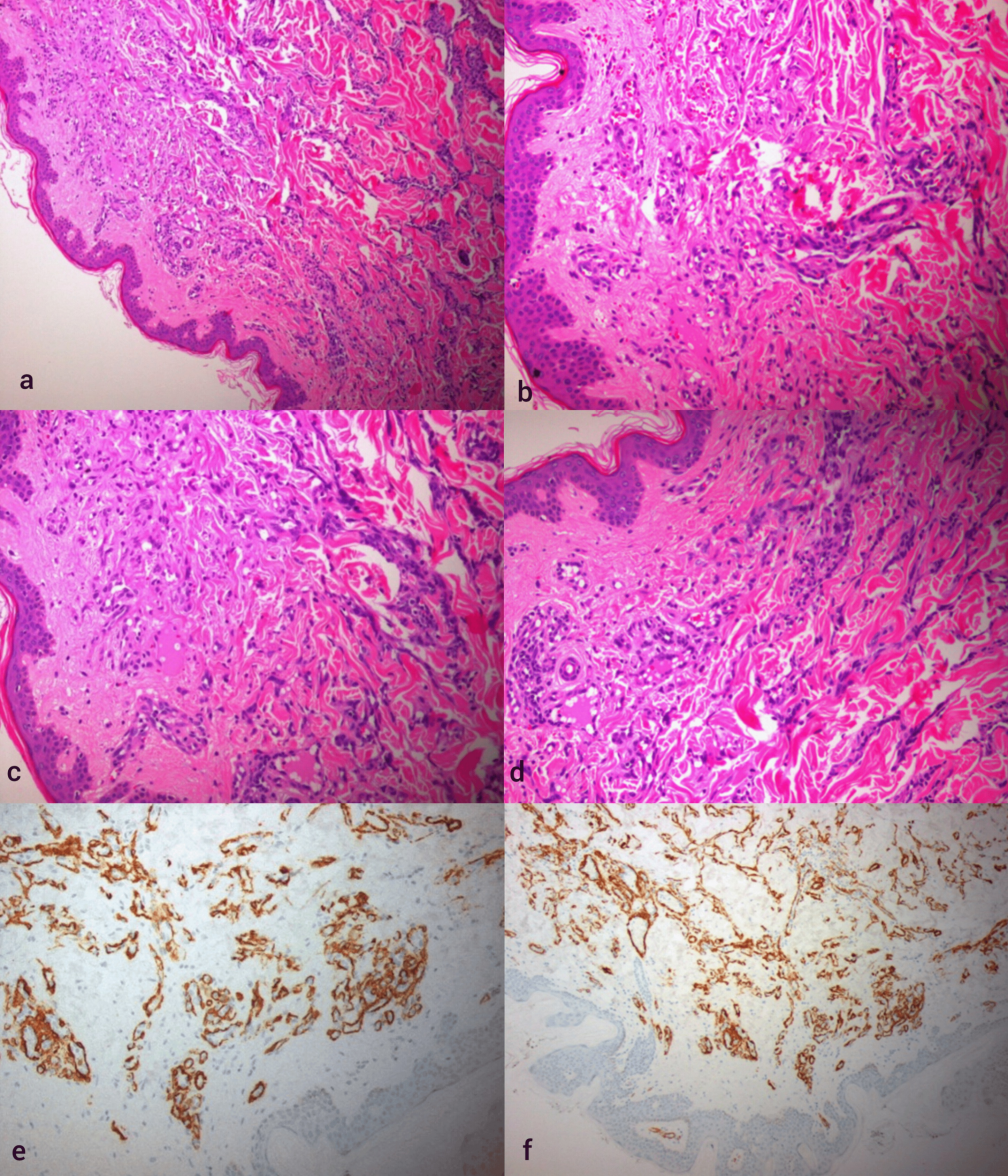
Histological and immunohistochemical aspects of the two cutaneous lesions a and b: Reactive angioendotheliomatosis shows dilated dermal and subcutaneous vessels disposed in diffuse, lobular, or mixed patterns. The latter vascular channels contain proliferations of small to enlarged endothelial cells that variably fill and often occlude vascular lumina (hematoxylin and eosin staining, ×100 and ×200 magnification). c and d: Reactive angioendotheliomatosis: seemingly disorganized arrangement of regular capillary vessels throughout the upper reticular dermis. This vascular growth pattern is often mistaken for angiosarcoma (hematoxylin and eosin staining, ×200 magnification). e and f: SMA membranous marker indication in the pericytic space surrounding the capillaries (×200 magnification). SMA: smooth muscle actin

A 46-year-old man was referred to our clinic with diffuse, progressively expanding red-purple plaques and subcutaneous nodules on the trunk and arms (Figure 1). A histopathological examination following the biopsy of two lesions was initially performed, and the results were compatible with reactive angioendotheliomatosis, featuring endothelial cell proliferation with papillary endothelial projections (Figures 2a-2d). Immunophenotyping was positive for smooth muscle actin (SMA), erythroblast transformation specific regulated gene 1 (ERG), CD31, and CD34 (Figures 2e, 2f). A quantitative DNA PCR for human herpesvirus 8 (HHV-8), pathognomonic for Kaposi sarcoma, yielded negative results. The endothelial cells did not express D2-40, a common lymphatic differentiation immunostaining marker.

At that point, there was no medical history of internal malignancy. However, in view of histopathological and clinical features that raised suspicion of an underlying disorder, the patient was subjected to an extensive computed tomography workup that detected an exceptionally large retroperitoneal mass, measuring 12.7 × 2.7 cm, which encased part of the right kidney. The patient underwent excision of the mass in the retroperitoneum with concomitant right radical nephrectomy.

**Figure 3:**
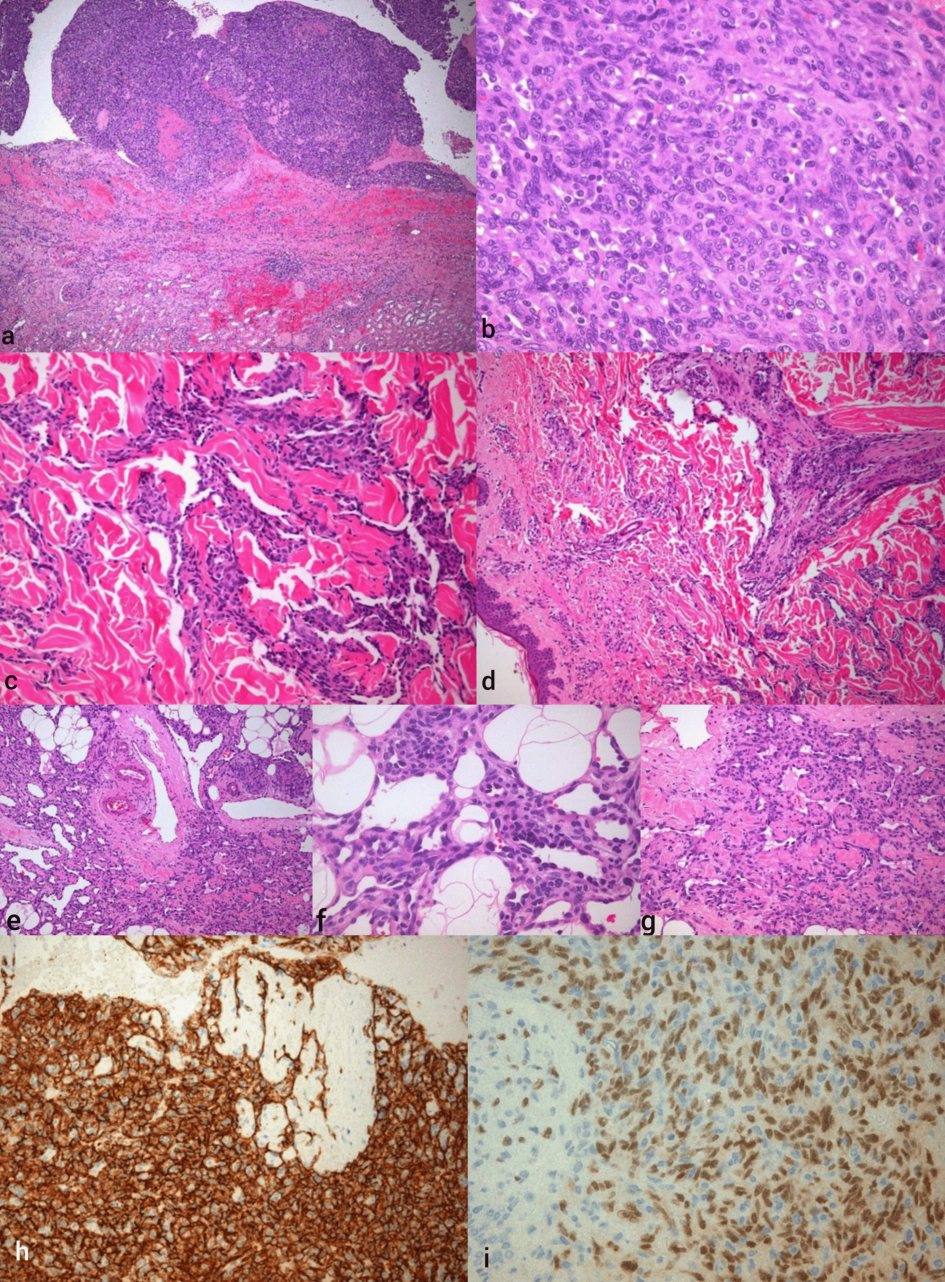
Histological and immunohistochemical findings of renal and cutaneous angiosarcoma a: Angiosarcoma typically has ill-defined margins, and it varies in its degree of cytological atypia and architectural differentiation (×40 magnification). b: Renal angiosarcoma: a poorly differentiated tumor composed of sheets of epithelioid and spindle cells with amphophilic cytoplasm (×400 magnification). c and d: Cutaneous angiosarcoma: the dermis contains an ill-defined tumor composed of irregular vascular channels lined by hyperchromatic, moderately atypical vascular endothelial cells. There is a mild surrounding chronic inflammatory infiltrate (hematoxylin and eosin). e, f, and g: Well-moderately differentiated angiosarcoma invading the reticular dermis. This lesion shows well-formed vascular spaces lined by mildly atypical flattened endothelial cells (hematoxylin and eosin). h: Angiosarcoma typically shows membranous CD31 positivity (×200 magnification). i: Nuclear immunoreactivity for ERG, the most sensitive and specific endothelial marker for angiosarcoma (×400 magnification). ERG: erythroblast transformation specific regulated gene 1

The histopathological examination supported the diagnosis of a primary retroperitoneal angiosarcoma. The neoplasm was composed of sheets of epithelioid and spindle cells with amphophilic cytoplasm, ill-defined margins, a variety of cytological atypia, and mitotic activity (Figures 3a, 3b). After the excision of the primary tumor, the skin lesions progressed rapidly, in contradiction to the expected clinical course of a putative reactive angioendotheliomatosis. As a result, two additional tissue samples were collected from cutaneous lesions for pathological evaluation.

**Figure 4:**
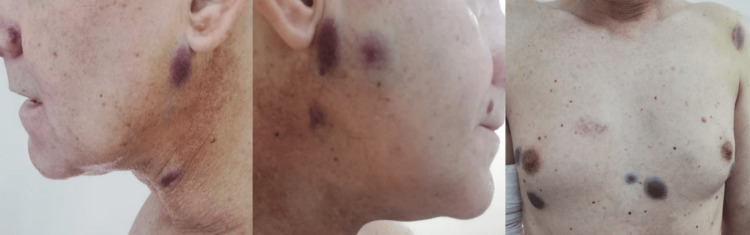
Progression of the skin lesions in size and number on the patient's trunk and face

Immunohistochemical staining was performed on the new skin specimens and resulted positive for CD31, CD34, ERG, friend leukemia integration 1 transcription factor (FLI1), and factor VIII, while antigen Kiel 67 (Ki-67) proliferation index was high, measuring 70% (Figures 3h, 3i). Fluorescence in situ hybridization (FISH) for myelocytoma (MYC) amplification was negative (Figures 3c, 3d). Considering the overview of immunohistological findings and FISH, the diagnosis of cutaneous metastasis of angiosarcoma was established (Figures 3e-3g). Concerning molecular analysis, single nucleotide and copy number genetic variants (SNVs/CNVs) were detected and classified as Tier II (possibly clinically significant) in the analyzed DNA material, as well as a low Total Mutation Burden (TMB) and a negative Microsatellite Instability Index (MSI). The analysis of 55 hybrid genes (RNA fusions) and splice variants of mRNA maturation products was negative. Therefore, chemotherapy with a combination of doxorubicin and cyclophosphamide was initiated. After six cycles of chemotherapy, the lesions increased in number and size in subsequent follow-up examinations, while the repeated staging procedures revealed no further metastasis (Figure 4). An adjuvant immunotherapy was planned with the purpose of halting cutaneous disease progression. Unfortunately, one year after diagnosis, the patient died.

## Discussion

In the early stages of angiosarcomas, discrete histological alterations can mimic benign intravascular endothelial proliferation [12]. A common mimicker of angiosarcoma is reactive angioendotheliomatosis, a rare benign disorder featuring an intravascular proliferation of cells expressing endothelial immunohistochemical cell markers [13]. Histological findings of reactive vascular entities and benign vascular growths usually demonstrate a symmetrical proliferative activity with a regular growth pattern consisting of lobules in capillaries and venules with regular endothelia in contrast to angiosarcomas, which are characterized by abnormal mitoses and atypically proliferating endothelia [11,14]. In our case, the initial skin lesions met no morphological or architectural criteria to be diagnosed as angiosarcoma. However, the clinical course and staging findings led us to be skeptical about the initial histological features. The histological findings aligned with our clinical suspicion only after repeated biopsies at a later stage, when the tumor architecture transformed toward a more disorganized vascular growth pattern with irregular vascular channels lined by hyperchromatic, moderately atypical vascular endothelial cells.

At this point, immunohistology was more than valuable. Although there is no pathognomonic immunohistopathological profile, angiosarcomas stain positive for at least one of the following endothelial cell markers: CD34, CD31, ERG, FLI1, and factor VIII-related antigen [13]. At the first histopathological examination, endothelial cells stained positive for CD31, CD34, and ERG, while in the second examination, all five endothelial markers stained positive. ERG is a highly specific marker for benign and malignant vascular proliferations [15]. Among the markers mentioned above, CD31 is the most sensitive and the most specific endothelial cell marker for angiosarcoma, while CD34 is expressed in most angiosarcoma cases. However, CD31 and CD34 stain positive in both angiosarcoma and reactive angioendotheliomatosis [1,5,14]. The fact that these two markers are also expressed in reactive angioendotheliomatosis reveals the vascular nature of the disease and a histological overlap between the two entities, which may lead to misdiagnosis. Further analysis of tumor cells for myelocytoma (MYC) amplification, a highly specific and sensitive diagnostic assay for secondary angiosarcomas, was negative. However, the lack of amplification of MYC does not suffice to rule out angiosarcoma. In these cases, Ki-67 could be a particularly useful diagnostic tool to distinguish angiosarcoma from other vascular benign entities [16]. In our case, the Ki-67 proliferation index of 70% is a strong indicator of the diagnosis of angiosarcoma.

Hereby, we must take into consideration the histological difficulties in diagnosing visceral angiosarcomas and their secondary cutaneous metastases. In contradiction to our findings, visceral angiosarcoma seems to be even more challenging to diagnose, being a great mimicker of benign intravascular endothelial-derived entities, due to low grade of tumor tissue aberration and a varying immunohistopathological profile. For example, the negativity of D2-40 (monoclonal antibody to M2A oncofetal antigen) and pericytic staining for SMA (alpha-smooth muscle actin), as in our case, can be found in visceral angiosarcoma, in contrast to primary cutaneous angiosarcoma [17].

## Conclusions

To conclude, the combination of clinical and histopathological clues is particularly important to establish the diagnosis of angiosarcoma, especially for well-differentiated and visceral angiosarcomas. Lacking a standard immunohistological pattern, angiosarcoma was initially masquerading as reactive angioendotheliomatosis of the skin. The positive staining for CD31, CD34, and ERG in combination with marked proliferation of the tumor, as expressed by proliferation index Ki-67, and the trajectory of the clinical course were the pointers to the correct diagnosis. The rapidly progressive nature of the skin lesions after the eradication of the causal factor fails to support the initial diagnosis of reactive angioendotheliomatosis and strongly indicates a malignancy, while the hypothesis of a coincidental simultaneous occurrence of two exceedingly rare diseases (angiosarcoma and reactive angioendotheliomatosis) seems implausible.
